# Longitudinal assessment of oral and gut microbiome overlap in patients with Alcohol Use Disorder undergoing inpatient treatment

**DOI:** 10.3389/fcimb.2025.1681781

**Published:** 2025-11-19

**Authors:** Jennifer J. Barb, Alexandria N. Hughes, Shubhi Nanda, Ralph Thadeus S. Tuason, Gwenyth R. Wallen, Katherine A. Maki

**Affiliations:** Translational Biobehavioral and Health Promotion Branch, Clinical Center, National Institutes of Health, Bethesda, MD, United States

**Keywords:** oral microbiome, gut microbiome, alcohol use disorder, AUD, Aitchison

## Abstract

Alcohol Use Disorder (AUD) is a condition associated with compulsive alcohol consumption and disruption across multiple physiological systems. This brief report builds on previously published research separately examining longitudinal changes in the oral and gut microbiomes of treatment-seeking individuals with AUD. Twenty-two participants diagnosed with severe AUD were enrolled in an inpatient treatment protocol (NCT02231840) and provided oral and stool samples over 28 days (goal 10 samples/participant). The aim of this brief report was to explore within-person overlap and compositional similarity of the oral and gut microbiomes at the genus level, using the Sorenson-Dice Index and Robust Aitchison Distance. Results indicated that the oral and gut microbiomes became less similar during the first week of treatment, with both the number of shared genera and Sorenson-Dice Index values decreasing significantly (*p* <.001). However, the Robust Aitchison Distance also decreased over time (*p* <.05), suggesting increased compositional similarity among the shared genera. These findings suggest early divergence of oral and gut microbiota during AUD treatment, where individuals were abstinent of alcohol, followed by stabilization of overlapping communities. This study highlights dynamic shifts in microbiome structure during a period of abstinence and underscores the importance of evaluating site-specific and cross-site microbial changes in AUD populations.

## 1 Introduction

Alcohol Use Disorder (AUD) is a persistent disorder characterized by the compulsive intake of alcohol, resulting in considerable disruption in an individual’s activities of daily living (Grant et al., 2015). Individuals with AUD are often at higher risks of developing several other comorbid health issues in addition to already pre-existing physical and mental co-morbid disorders that may be associated with AUD. The Diagnostic and Statistical Manual of Mental Disorders (DSM-5) diagnosis of AUD is based on various criteria, such as cravings, failed efforts to reduce alcohol consumption, and persistent use despite negative repercussions (Grant et al., 2015). This condition impacts various physiological systems, including the gastrointestinal tract, where it can disturb the equilibrium of both oral and the gut microbiomes of people with AUD.

The gut microbiome, comprising a complex assemblage of bacteria inhabiting the digestive tract, is essential for sustaining gut health and contributes to overall health and wellness (Madhogaria et al., 2022; Koutromanos et al., 2024). Research has shown that alcohol use can modify the composition and functionality of the gut microbiome, which may contribute to AUD symptoms and can potentially be detrimental to recovery from the disease (Ames et al., 2020). A study published in 2024 showed that people with AUD frequently display a unique gut microbiome profile, marked by dysbiosis, and decreased microbial diversity (Piacentino et al., 2024). Alcohol use has also shown to cause disruptions to the gut microbiome, having more gastric and anaerobic bacteria in the colon, which further can cause irritation to the gut health of people with AUD (Bode et al., 1984). Another study in 2024 showed increased intestinal permeability and reduced resilience of the intestinal barrier in individuals with alcoholic liver disease, further emphasizing the detrimental impact to gut health in the context of alcohol consumption (Leclercq et al., 2014; Swanson et al., 2024).

Not only is the gastrointestinal tract disrupted with chronic alcohol use, but individuals with AUD also often have poor oral hygiene with a higher prevalence of periodontal disease (Manicone et al., 2017). The oral microbiome is the second most diverse and largest of the human microbiome niches after the gut microbiome, which when it is in dysbiosis can cause higher health risks, such as seen in a literature review assessing relationships between the oral microbiome of smokers and higher cardiovascular risk (Deo and Deshmukh, 2019; Maki et al., 2022). Previous research has shown that the oral microbiome was disrupted, and a higher number of pathogenic genera was seen with chronic alcohol use (Li et al., 2022). Dysbiosis of the oral microbiome can lead to periodontal disease, tooth decay and loss, and oral mucosal lesions, which can complicate nutrient breakdown and overall health which can be an additional comorbid condition experiences by individuals with AUD (Gandhi et al., 2024).

**Figure 1:**
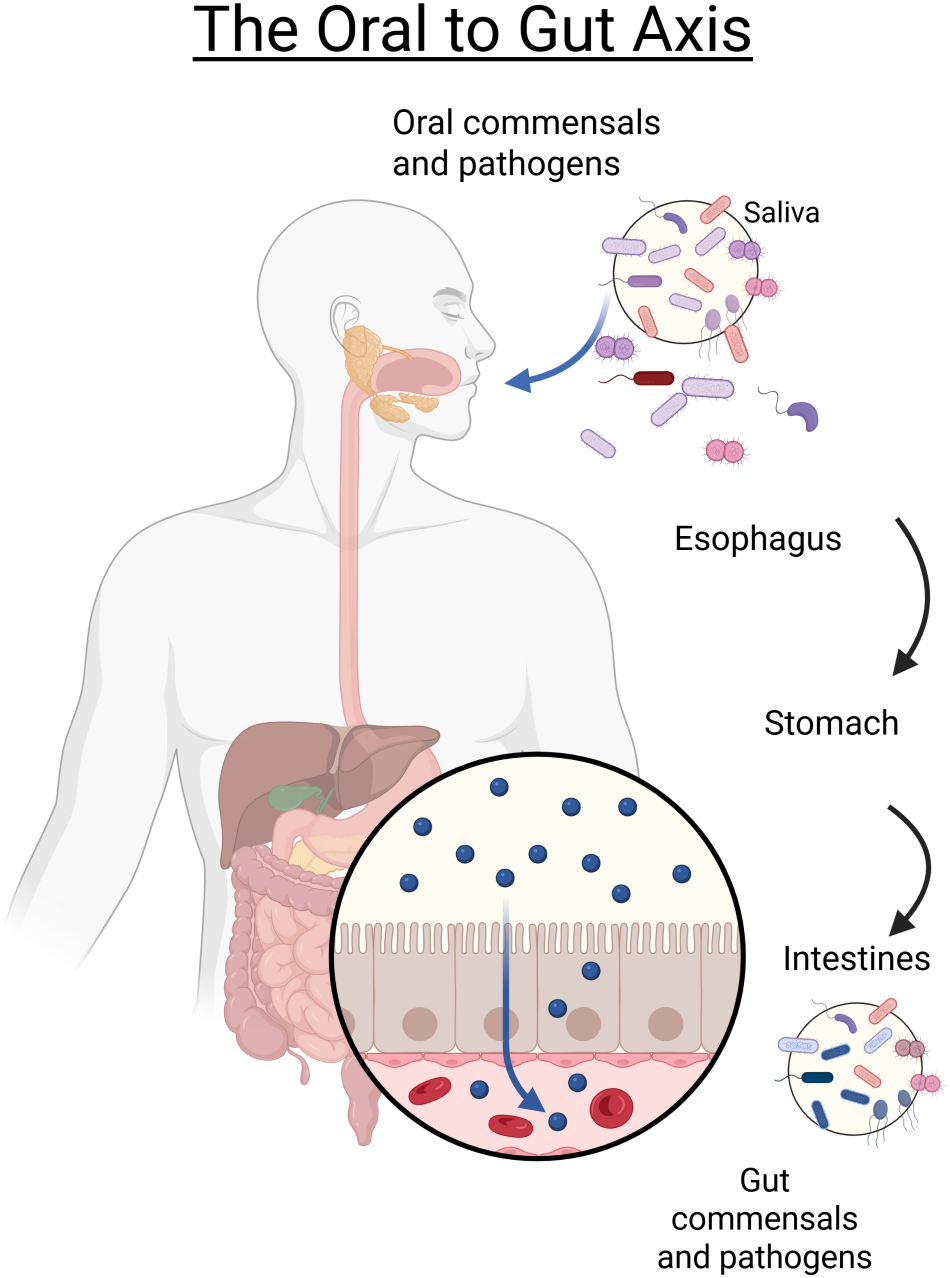
Graphical representation of the oral to gut axis. Bacteria from the oral cavity can migrate via saliva through the gastrointestinal tract, with gastric acid and bile acting as selective barriers. In the gut, a diverse microbial community interacts with the host immune system at the mucosal interface. Bidirectional links exist: oral dysbiosis can promote gut dysbiosis, while gut inflammation can reciprocally alter oral communities. These disruptions contribute to diseases including colorectal cancer, inflammatory bowel disease, and metabolic disorders. Alcohol use further disrupts both oral and gut microbiota, leading to periodontal disease, caries, gingivitis, and gut inflammation. Chronic alcohol use and Alcohol Use Disorder (AUD) exacerbate these disruptions. Figure created in biorender.com

The intersection of the oral and gut microbiomes is an under researched area despite their link at the start of the digestive track through the end of the digestion (**Figure 1**), though, several clinical studies have examined this connection in specific patient populations. Foundational work has demonstrated that oral microbes can translocate and establish within the gut environment, with implications for both microbial community structure and host metabolism. For example, Schmidt et al. showed extensive transmission of oral taxa to the gut across diverse human cohorts, supporting the concept of an oral–gut microbial axis (Schmidt et al., 2019). In fact, the authors of this work developed a list of strain level taxa that were reported to be ‘*transmitter’* taxa between the oral and gut microbiota (Schmidt et al., 2019). In murine models, oral administration of *Porphyromonas gingivalis* altered both gut microbial composition and the host serum metabolome, providing mechanistic evidence of how oral dysbiosis can impact gut and systemic physiology (Kato et al., 2018). More recently, integrated microbiome–metabolome studies have further linked oral dysbiosis with downstream gut microbial and metabolic shifts in human populations, including those with periodontitis (Dong et al., 2022; Cheng et al., 2024). These studies collectively establish a foundation for considering the oral and gut microbiomes as interconnected ecosystems whose disruption may exacerbate disease processes.

A 2024 study investigated the interplay between oral and gut microbiomes in pre-clinical mouse experiments and in samples collected from patients with inflammatory bowel disease and healthy controls to compare their collective impact on patient outcomes (Liao et al., 2024). Similarly, another study examined the oral and gut microbiomes among men with and without human immunodeficiency virus, where distinct microbial profiles at the genus level between the oral and gut niches were observed, which further diverged following antiretroviral therapy (Li et al., 2023). Compared to HIV-uninfected controls, men with acute and chronic HIV diagnoses exhibited persistent reductions in oral microbiome diversity and genus-level shifts distinct from those observed in the gut microbiome (Kato et al., 2018). Another similar study was conducted on a population with acute leukemia where the authors drew parallels on associations between increased oral and gut microbiome community similarity and more frequent adverse clinical outcomes including an increased rate of infection-related complications (Franklin et al., 2022).

While these studies offer important insights within specific clinical populations, a more comprehensive understanding of the oral–gut axis has also emerged through broader literature reviews (Elghannam et al., 2024; Kunath et al., 2024). One of these reviews found that oral microbiome dysbiosis, including the translocation of oral bacteria to the gut, has been implicated in various gastrointestinal conditions such as colorectal cancer and inflammatory bowel disease (Elghannam et al., 2024). Another review reported that the oral–gut microbiome axis may play a significant role in disease development, as oral microbes are more likely to colonize the gut under periods of altered homeostasis and instability to disrupt host-microbe interactions (Kunath et al., 2024). In a 2020 literature review conducted by several of the authors of this work, we examined similarities and differences between the oral and gut microbiome communities in healthy human studies, highlighting increased bacterial overlap in the mouth and gut occurs when taxa are annotated at the level of species strain, suggesting the rates of oral to gut translocation may change as technology advances and bacterial annotation becomes more precise (Maki et al., 2021). Our review suggested that changes in one microbiome may influence the other, reinforcing the importance of considering multiple bacterial microbiome niches to gain a complete picture of health implications in both clinical and therapeutic contexts (Li et al., 2023). Together, these prior studies provide a framework for this brief exploratory analysis, which builds on our earlier work in newly abstinent individuals with AUD and addresses the need for more research on oral–gut microbiome similarities and differences across clinical contexts (Ames et al., 2020; Barb et al., 2022). The aim of this brief report is to examine bacterial genera shared between the oral and gut microbiomes and to evaluate changes in their compositional similarity over time using longitudinal samples from individuals with AUD undergoing inpatient treatment and alcohol abstinence.

## 2 Methods

### 2.1 Sample description

This brief report builds upon previously published research investigating longitudinal changes of the oral and gut microbiomes of treatment seeking individuals with alcohol use disorder (Ames et al., 2020; Barb et al., 2022). Briefly, the sample population consisted of 22 individuals with AUD, all of whom met Diagnostic and Statistical Manual of Mental Disorder-5 criteria severe AUD (American Psychiatric Association, 2022). Participants were enrolled in the NIAAA Natural History Protocol (NCT02231840) and provided consent for participation in a longitudinal observational protocol to evaluate the impact of heavy alcohol use (on admission) and abstinence from alcohol over 27 days on bacterial communities of the oral and gut microbiomes (protocol NCT02911077) as previously described (Ames et al., 2020; Barb et al., 2022). Alcohol consumption was assessed using the 90-day Alcohol Timeline Followback (TLFB) which details self-reported information on daily alcohol use over the past 90 days prior to their admission into the inpatient treatment program (Sobell and Sobell, 1992). Data collected included quantity and frequency of alcohol consumption, allowing for calculation of total drinks, average drinks per drinking day, and percent days abstinent from alcohol in the prior 90 days, and detailed alcohol intake profiles and clinical phenotyping of the study participants were reported in the primary papers (Ames et al., 2020; Barb et al., 2022). Alcohol choice and dose categorization has been previously described; a brief summary is included in the **Supplementary Material**. Periodontal disease status of all participants was assessed during a dental and periodontal examination by dental health professionals as previously described (Barb et al., 2022). Periodontal disease was classified as none, mild, moderate, or severe in this study cohort.

### 2.2 Description of sequencing and bioinformatics

A full description of the sequencing, processing and bioinformatics details are available in the two primary publications (Ames et al., 2020; Barb et al., 2022) and also in the **Supplementary Material**. Briefly, DNA was extracted from all samples, and the 16S rRNA gene was amplified using the Ion 16S™ Metagenomics Kit (ThermoFisher), targeting seven hypervariable regions, followed by sequencing on the Ion Torrent S5 platform. Samples were processed using standardized protocols including homogenization, centrifugation, and storage at −80°C prior to sequencing. Sequence reads were filtered, clustered or denoised into OTUs/ZOTUs, assigned taxonomy using SINTAX with the RDP reference database, and summarized at the genus level across hypervariable regions. Oral and gut genus-level datasets were filtered to retain genera present in ≥25% of samples and merged, resulting in a combined dataset of 159 genera for cross-site microbiome analyses, with average counts across all timepoints shown in **Supplementary Table 2**.

### 2.3 Description of datasets

This study is a secondary analysis examining within-subject oral and gut microbiome features over time in individuals with AUD. Oral (tongue brushing) and gut (fecal) microbiome samples were collected daily for the first week of inpatient admission (days 1-7) and then weekly for the following three weeks (beginning on week 2, week 3, and week 4). The oral and gut microbiome data analyzed in this report have been previously independently described in separate publications (Ames et al., 2020; Barb et al., 2022); this report extends the primary work by investigating the within individual overlap of genera between the oral and gut microbiomes. Total genus counts for each sample at each timepoint were merged and used for the current analysis. Genera counts will be reported in this study. For full sample collection and processing procedures see primary publications and **Supplementary Material**. Oral and gut count values were averaged over patients at each timepoint to create heatmaps, and the number of patients with shared genera in the oral and gut microbiomes at each timepoint was tabulated by presence (nonzero count) in both samples. Composition of individual oral and gut microbiota samples were evaluated by the Shannon alpha diversity index to measure the richness and evenness of individual bacterial communities over time. To evaluate compositional similarity and differences between oral and gut microbiome samples within individuals over time, beta diversity was assessed using two metrics: the Sorenson–Dice Index, which captures genus-level similarity across sites based on presence/absence data, and the Robust Aitchison Distance, which quantifies compositional dissimilarity among the shared genera annotated in the oral and gut microbiomes (Zou et al., 2004; Martino et al., 2019). For the bioinformatics sample pre-processing of the microbiome samples and the merging across oral and gut microbiome datasets, see **Supplementary Material**.

### 2.4 Statistical analysis

Longitudinal changes in Shannon Diversity Index (SDI) values were tested using generalized additive models within the oral and gut samples. Differences between oral and gut microbiome SDI values collapsed over time were tested using a two-sided T-test. Generalized additive mixed models (GAMMs) were fit to the Sorenson-Dice Index values and the Robust Aitchison values to model the nonlinear effect of time in these data while accounting for within-patient repeated measurements. These nonlinear trends were modeled with GAMMs to test the hypothesis that the trends significantly changed over time and the p-values of the smooth terms were reported. GAMMs were fit using the ‘mgcv’ package in R (4.3.1). Alcohol dose, choice and days since last drink were investigated with shared overlap metrics during the first week and generalized linear mixed models (LMM) were employed. For the number of shared genera, which is a count variable, a Poisson LMM was employed. See **Supplementary Material** for a full description of the methods. Statistical significance was set at the alpha = 0.05 level for all testing.

## 3 Results

### 3.1 Sample population

The patient demographics and clinical characteristics of the population was previously described in two primary papers assessing gut and oral microbiome of the patient population (Ames et al., 2020; Barb et al., 2022) (**Supplementary Table 1**). Briefly the current analysis includes 22 individuals with AUD who provided oral and gut microbiome samples through the course of their 4-week inpatient treatment. Most of the cohort were males (63.6%), with average age of 46 years (45.82 ± 13.0), and normal BMI (23.87 ± 2.55). Seventeen of the 22 participants had moderate or severe periodontal disease, and the majority were current smokers (73%, n=16). Based on the 90-day TLFB, participants consumed an average of 16.20 ± 10.59 drinks per drinking day and 76.57 ± 22.51 heavy drinking days (Ames et al., 2020; Barb et al., 2022). Previously reported alcohol dose and choice type showed that 8 (36.4%) were classified as low heavy drinkers (LHD) and 14 (63.6%) as very heavy drinkers (VHD). Reported typical alcohol preferences varied across the sample: 4 participants (18.2%) primarily consumed beer, 5 (22.7%) reported both beer and liquor, 6 (27.3%) consumed liquor, and 7 (31.8%) reported wine as their typical alcoholic beverage (**Supplementary Table 4**) and the average days since last drink before treatment was -1.59 ± 4.85.

### 3.2 Oral and gut microbial genus counts, overlap and alpha and beta diversity

Genera abundance count tables were downloaded from previous publications for all participants for the oral and gut microbiome datasets (Ames et al., 2020; Barb et al., 2022). Average counts for oral and gut microbiome samples across all sampling timepoints, are shown in **Table 1** and **Supplementary Table 2**. Of the up to ten samples (oral and gut) collected for each participant, **Table 2** shows the number of participants (out of 22) who had complete gut and oral microbiome samples collected at each timepoint. The number of participants with matched oral and gut samples at the same timepoint ranged from 4 participants on day 1 to 18 participants on day 5 and weeks 2-4.

**Table 1:** Average genera and alpha and beta diversity across all timepoints.

Number of genera or diversity metric	Oral or gut	Mean (SD) across all timepoints
Number of Genera	Gut	47.32 (7.47)
	Oral	43.19 (9.10)
	Shared (Gut and Oral)	9.60 (3.91)
Shannon Diversity Index	Gut	2.58 (0.26)
	Oral	2.10 (0.35)
Sorenson-Dice Index		0.21 (0.06)
Robust Aitchison Distance		12.20 (2.38)

Shannon index assessed within oral and gut microbiome separately.

**Table 2:** Average genera and alpha and beta diversity for each timepoint.

Sample timepoint		Day 1	Day 2	Day 3	Day 4	Day 5	Day 6	Day 7	Week 2	Week 3	Week 4
# of subjects with both oral and gut sample collected		4	15	16	17	18	14	16	18	18	18
Mean (SD)											
# of Genera	Gut	46.00 (5.10)	45.13 (8.74)	47.12 (9.23)	46.65 (8.08)	48.78 (7.37)	45.43 (7.17)	47.75 (6.77)	48.17 (7.37)	48.00 (7.37)	48.33 (6.56)
	Oral	45.50 (18.73)	44.73 (7.14)	44.88 (12.02)	42.82 (7.88)	43.67 (6.85)	44.21 (7.83)	41.88 (8.52)	41.11 (10.59)	42.39 (8.73)	43.00 (9.76)
# of Genera shared between oral and gut		15.25 (8.02)	10.33 (2.29)	11.75 (7.18)	8.82 (3.03)	8.50 (2.36)	9.36 (2.82)	8.12 (2.22)	9.11 (4.46)	9.39 (2.77)	9.89 (2.81)
Shannon Diversity Index (Alpha Diversity)	Gut	2.65 (0.07)	2.49 (0.26)	2.56 (0.28)	2.62 (0.28)	2.61 (0.21)	2.51 (0.25)	2.60 (0.25)	2.64 (0.30)	2.54 (0.25)	2.60 (0.25)
	Oral	1.96 (0.39)	2.17 (0.26)	2.20 (0.36)	2.11 (0.36)	2.17 (0.35)	2.02 (0.38)	2.09 (0.34)	2.07 (0.43)	2.13 (0.30)	2.01 (0.34)
Sorenson-Dice Index (Beta Diversity)		0.32 (0.11)	0.23 (0.04)	0.24 (0.09)	0.20 (0.06)	0.18 (0.04)	0.21 (0.05)	0.18 (0.05)	0.20 (0.07)	0.21 (0.05)	0.21 (0.05)
Robust Aitchison Distance (Beta Diversity)		15.12 (3.47)	12.58 (2.73)	12.82 (2.14)	12.16 (2.89)	11.81 (1.89)	12.55 (1.83)	10.86 (1.55)	11.56 (3.00)	12.07 (2.25)	12.79 (1.83)

**Figure 2:**
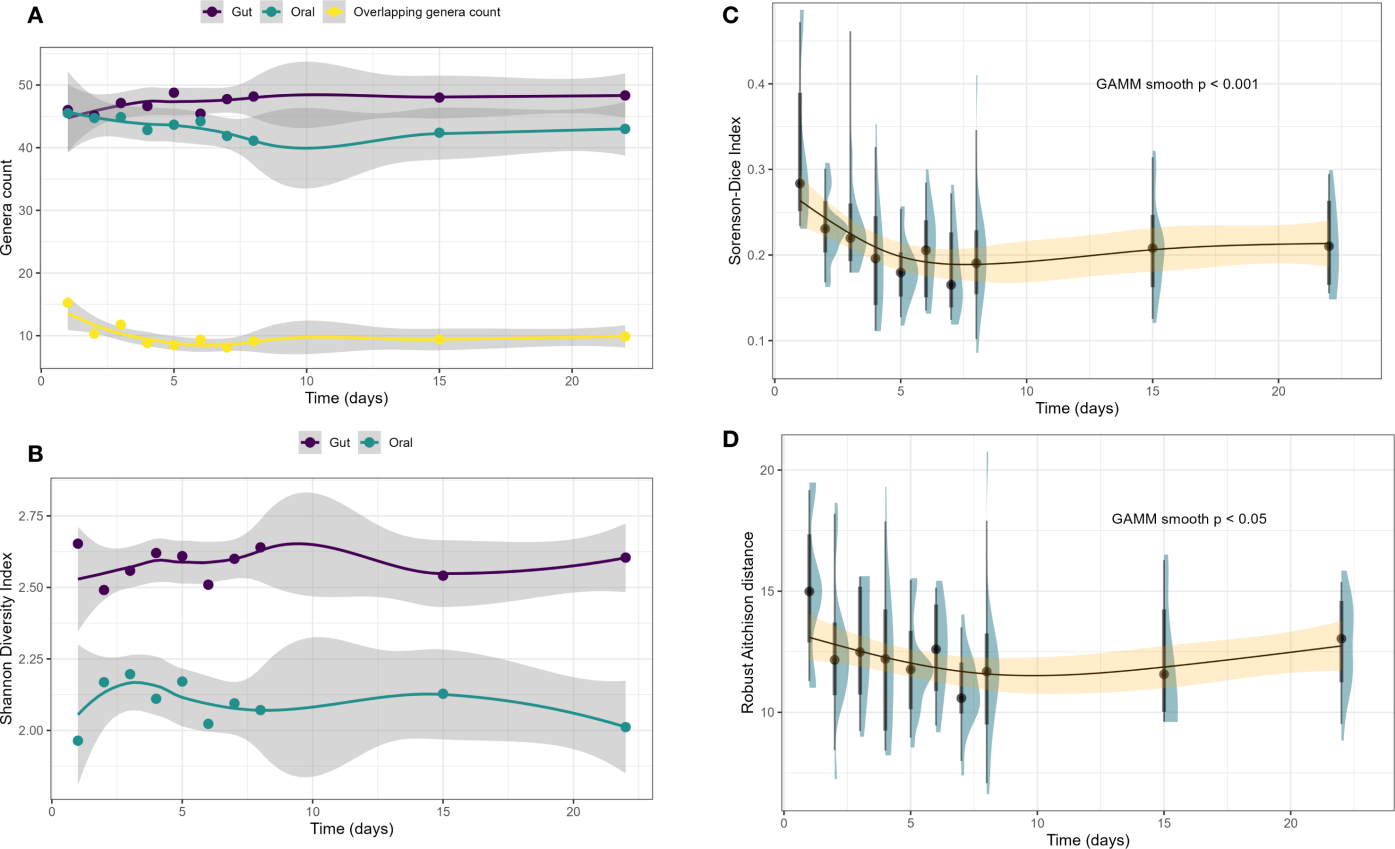
Temporal shift in oral–gut microbiome overlap and shared genus compositional structure. **(A)** Number of genera in the oral (teal) and gut (purple) microbiomes over time, along with the number of overlapping genera shared between both sites (yellow). **(B)** Shannon Diversity Index for oral and gut microbiomes over time, showing consistently higher alpha diversity in the gut across all timepoints. **(C)** Sorenson-Dice Index, a measure of genus-level similarity between oral and gut samples, significantly decreased over time (GAMM p < 0.001), with the most rapid decline during the first week, indicating reduced oral–gut overlap. **(D)** Robust Aitchison distance, representing compositional dissimilarity among shared genera, also declined over time (GAMM p < 0.05), suggesting increasing similarity in the structure of shared taxa despite overall reduced overlap. Shaded regions in **(A, B)** are standard errors of the smoothing technique (LOESS) applied to the point estimates shown; shaded regions in **(C, D)** and represent 95% confidence intervals around smoothed GAMM trend lines.

The average number of genera detected across all samples was 47.32 ± 7.47 for the gut and 43.19 ± 9.10 for the oral microbiome (**Figure 2A**). On average, 9.60 ± 3.91 genera were shared between the oral and gut microbiomes across timepoints. Alpha diversity, as measured by the Shannon Diversity Index, was higher in the gut (2.58 ± 0.26; range: 2.49–2.65) than in the oral microbiome (2.10 ± 0.35; range:1.96–2.20) across all samples (**Figure 2B**, **Table 1**) and when comparing the average over all timepoints, a significance average SDI between oral and gut was observed (*p<.001*) (**Supplementary Table 3**). Beta diversity measuring similarity (overlap) between the paired oral and gut samples was 0.21 ± 0.06 (Sorenson-Dice Index) which indicated a relatively low overlap. The average compositional distance within individuals between the two sites (Robust Aitchison Distance) was 12.20 ± 2.38.

### 3.3 Assessment of oral-to-gut overlap and similarity at each timepoint during inpatient treatment

Oral and microbiome feature metrics at each of the ten sampling timepoints across all patients during the inpatient stay are shown in **Table 2**. The average number of oral genera detected showed slight variability over time (range: 41.11–45.50), while the gut remained relatively stable across timepoints (range: 45.13–48.78) (**Figure 2A**). The number of genera shared between oral and gut samples was highest on day 1 (15.25 ± 8.02), then declined and stabilized across later timepoints (range: 8.12–11.75) (**Figure 2A**). A depiction of the number of shared genera between the oral and gut microbiome at day 1 and then at weeks 2–4 is shown in **Supplementary Figure 1**. No significant linear alpha diversity changes were observed in either the oral or gut microbiomes over time (p>.05) (**Figure 2B**). When SDI values were compared at each timepoint between the oral and gut microbiomes, all timepoints were significant (all *p<.018*) (**Supplementary Figure 2**, **Supplementary Table 3**).

The Sorenson-Dice Index, measuring beta diversity and genus level overlap between the microbiome niches, decreased significantly over time, dropping from 0.32 ± 0.11 on day 1 to approximately 0.23 ± 0.04–0.21 ± 0.05 by week 2 through week 4 (*p* < .001; **Figure 2C**). This trend indicates a reduction in shared genera between the oral and gut microbiomes, particularly during the first week. In contrast, the Robust Aitchison Distance, also showed a significant decrease over time (p = .039; **Figure 2D**), declining from 15.12 ± 3.47 on day 1 to a narrower range of 10.86 ± 1.55–12.79 ± 1.83 in later weeks which suggests an increase in compositional similarity of shared oral and gut genera.

### 3.4 Assessment of oral-to-gut overlap by past alcohol dose, choice and consumption variables

When alcohol dose and choice type was explored in week 1 among the Sorenson-Dice index, VHD compared to LHD had a higher Sorenson-Dice index (p = 0.013), and a preference for alcohol types beer and liquor (p = 0.03), liquor (p = 0.002), or wine (p = 0.001) had lower Sorenson-Dice indices compared to those who preferred beer only (**Supplementary Table 5**). For the Robust Aitchison Distance, which was distributed roughly symmetrically, we fit an LMM and found that in contrast to the analyses for Sorenson-Dice and number of shared genera, there was no significant relationship between Robust Aitchison Distance and alcohol dose or choice during the first week (**Supplementary Table 5**).

Similarly, VHD compared to LHD had a higher number of shared genera (p = 0.006), and a preference for liquor (p = 0.006) or wine (p = 0.006) had lower numbers of shared genera compared to those who preferred beer only (beer and liquor vs beer was not significant) (**Supplementary Table 5**). Additionally, no significant relationships were observed between days since last drink and past average drinks/days with any of these measures (p>.05) (**Supplementary Table 6**).

### 3.5 Assessment of genus level counts in the oral and gut microbiomes and the proportional presence between the niches within patients

**Figure 3:**
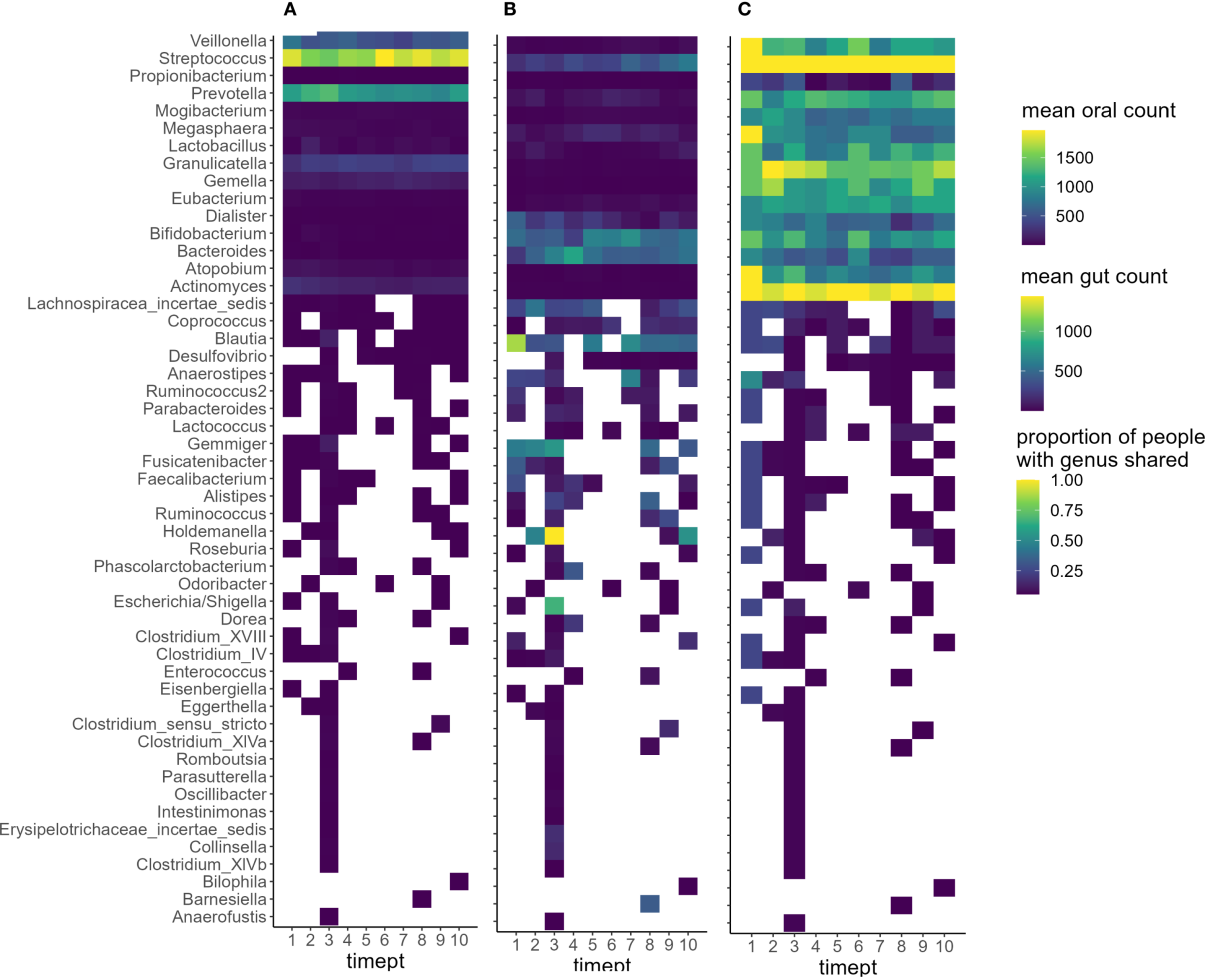
Shared genus level counts in oral and gut microbiome and the proportion of shared genera across individuals during inpatient treatment. Legend: Shared genera across all timepoints and limited to genera co-occurring at any timepoint. **(A)** Oral microbiome genus-level counts across ten timepoints. **(B)** Gut microbiome genus-level counts across ten timepoints. Color intensity indicates abundance (count values). **(C)** Proportion of participants sharing each genus in both oral and gut samples at each timepoint. Brighter colors (yellow) indicate genera shared by a larger proportion of individuals.

To examine temporal trends in oral-gut microbiome overlap and the presence of shared genera, data from all participants were visualized across ten timepoints (**Figure 2**). The oral microbiome average counts (**Figure 3A**) were dominated by genera such as *Granulicatella* (346.71 ± 49.76), *Prevotella* (1065.67 ± 143.71), *Streptococcus* (1746.93 ± 156.76), and *Veillonella* (542.14 ± 102.68), all of which are well-established oral commensals commonly found in the human mouth. The gut microbiome (**Figure 3B**) was characterized by typical gut-associated genera including *Bacteroides* (494.93 ± 161.87), *Bifidobacterium* (506.88 ± 131.16), *Blautia* (590.36 ± 281.99), *Gemmiger* (574.58 ± 139.79), and *Holdemanella* (710.33 ± 571.40). When assessing the overlap of the presence of oral and gut shared genera within individuals (**Figure 3C**), a large proportion of individuals shared *Streptococcus* (100%), *Actinomyces* (97.56%), *Granulicatella* (81.45%), *Gemella* (65.33%), and *Veillonella* (64.37%) in the oral and gut microbiome communities across all timepoints.

## 4 Discussion

This brief report presents an exploratory analysis assessing the dynamic relationship between the oral and gut microbiomes during early abstinence among treatment seeking individuals with AUD. Building on prior work that evaluated these microbiomes independently, we explored genus-level overlap and compositional similarity within individuals across ten longitudinal timepoints during a four-week inpatient treatment period for AUD (Ames et al., 2020; Barb et al., 2022). While the oral and gut microbiomes remained relatively stable in terms of within-site diversity, we observed a marked decline in the number of shared genera and Sorenson-Dice Index values within the first week of abstinence with a statistically significant change (*p* < .001), followed by increased similarity among the shared genera as assessed by the Robust Aitchison Distance which also was significant (*p* = .0390). For the first time in this patient population, this report explored the similarities and differences of the oral and gut microbiomes of people with AUD and the findings highlight a dynamic and possible adaptive nature of the microbiome in response to alcohol cessation.

As shown by the longitudinal assessment of the Sorenson-Dice index, the early decline in the number of shared genera found might be due to site-specific restructuring of the microbiome in response to alcohol cessation and a new environment. Prior studies suggest that chronic alcohol consumption can disrupt gut barrier function and contribute to microbial dysbiosis (Leclercq et al., 2014), which may, in some cases, facilitate the translocation and persistence of oral bacteria in the gut. The greater oral-gut microbial overlap observed at admission, which occurred immediately after heavy and chronic alcohol use, followed by a decline over time, may reflect early restoration of oral health or gut barrier integrity and microbial niche separation during abstinence. Interestingly, previous work on this patient cohort demonstrated improvements in the Beck Oral Assessment Scale, a clinical measure of physical oral health, over the course of inpatient treatment, indicating a concurrent enhancement in overall oral health (Franklin et al., 2022). Supporting this, a 2019 study examining salivary and fecal microbial strains across a large international cohort demonstrated that migration and colonization of oral microbes on both the species and the strain level in the large intestine is common. The authors proposed that the gastrointestinal tract may be vulnerable to oral microbial translocation under both healthy and dysbiotic conditions which means that the oral-gut transmission is not only widespread in healthy individuals but elevated in individuals with a disease like colorectal cancer or rheumatoid arthritis. This indicates that gut microbes often originate from one’s own oral microbiome (Schmidt et al., 2019).

Despite a reduction in the number of shared genera, compositional similarity along the remaining shared genera increased over time, as indicated by a decreasing Robust Aitchison distance. This suggests of the shared bacteria (at the taxonomic level of genus) that were present in both oral and gut microbiomes after abstinence from alcohol, the abundance of the genera became more similar across microbial niches as inpatient treatment progressed. Notably, we identified a core group of genera, including *Streptococcus* (100%), *Actinomyces* (97%), *Granulicatella* (81%) *and Gemella* (65%), *Veillonella* (64%) and *Prevotella* (63%) that were consistently shared across the oral and gut microbiomes in a large proportion of participants. Schmidt et al. (2019) reported 74 strain-level taxa as “transmitter” bacteria between the oral and gut microbiomes (Schmidt et al., 2019). Comparing their list with the genera shared in at least 60% of our patients, we found that five of the six genera most consistently shared in our cohort (*Streptococcus, Actinomyces, Granulicatella, Gemella*, and *Veillonella*) overlapped with the ‘transmitter’ taxa described by Schmidt et al. This concordance supports the biological plausibility of oral–gut microbial exchange in AUD and situates our findings within prior work on oral–gut transmission. Furthermore, these genera are well-established constituents of the oral microbiota (Zaura et al., 2009), and their continued detection in the gut microbiome, although typically at lower relative abundance, may reflect a residual effect of translocation via swallowed saliva or adaptation to altered gut environments following chronic alcohol use.

While this work offers valuable hypothesis-generating observations and directions for future research, it is not without limitations. The relatively small sample size and absence of a control group without AUD limits the generalizability of our findings and reduces the power to detect more subtle microbial changes or conclude which changes are specific to AUD. Additionally, the diversity and complexity of the oral and gut microbiomes, combined with heterogeneity in human behaviors, may introduce confounding variables that were not fully accounted for. As the participants with AUD had certain comorbidities and had ongoing medication use, we cannot fully account for the effects that medications might have had on the oral or gut microbiome features. Medication use was not a primary aim of this work but was previously reported. We acknowledge that there could be an impact on the microbiome of the participants who were receiving medication during inpatient treatment. Furthermore, this study relied on 16S rRNA gene sequencing targeting multiple variable regions, enabling taxonomic resolution at the genus level, but not at the more specific species or strain level where increased oral to gut translocation has been more frequently observed. Future studies incorporating larger cohorts and shotgun metagenomic sequencing could provide more precise microbial identification and enable deeper understanding of microbiome dynamics across sites in both patients with AUD and across other clinical populations.

### 4.1 Clinical implications

Larger studies are needed to determine whether oral microbial profiles can reliably serve as a less-invasive proxy for certain abundant gut microbial taxa. Oral sample collection is simpler, less invasive, and generally more acceptable to participants compared to stool sampling, making it an attractive alternative for clinical or population-based microbiome research. Not to mention that most individuals may prefer to collect an oral sample than a stool sample. Identifying a consistent set of shared taxa between the oral and gut microbiomes that are differentially present at periods of alcohol use and abstinence, respectively, could provide a practical, non-invasive screening tool for detecting alcohol-related microbiome disruptions.

Although our findings suggest that the number of shared genera across oral–gut microbial niches decline during abstinence and that shared taxa may converge in abundance over time, further research is needed to assess whether these trends are robust across larger, more diverse cohorts. Moreover, studies integrating functional metagenomics and host biomarkers are essential to clarify the biological and clinical significance of these microbial shifts, particularly in relation to oral health, gut barrier integrity, systemic inflammation, and long-term recovery in individuals with AUD.

## 5 Conclusion

The observed shifts in oral–gut microbial overlap and compositional similarity during the first month of early abstinence in treatment-seeking individuals with AUD suggest dynamic restructuring of microbiome communities following alcohol cessation, potentially reflecting early stabilization of oral and gastrointestinal environments and reestablishment of site-specific microbial niches. Notably, while the number of shared genera declined over time, the increased compositional similarity among these shared taxa may indicate stabilizing microbial relationships during recovery. This exploratory study lays preliminary groundwork for assessing the oral microbiome as a non-invasive proxy for monitoring systemic microbial changes in the context of alcohol-related dysbiosis. Future research should build on these findings using larger, more diverse cohorts with matched controls, longitudinal sampling during extended periods of abstinence, and higher-resolution techniques such as strain-level metagenomics and functional profiling to elucidate the biological and clinical significance of oral–gut microbial similarities, differences and interactions during health and disease.

## Author’s note

This research was supported by the Intramural Research Program of the National Institutes of Health (NIH). The contributions of the NIH authors were made as part of their official duties as NIH federal employees, are in compliance with agency policy requirements, and are considered Works of the United States Government. However, the findings and conclusions presented in this paper are those of the authors and do not necessarily reflect the views of the NIH or the U.S. Department of Health and Human Services.

## Data Availability

The original contributions presented in the study are included in the article/**Supplementary Material**. Further inquiries can be directed to the corresponding author.
