# Supplementary material for: Longitudinal assessment of oral and gut microbiome overlap in patients with Alcohol Use Disorder undergoing inpatient treatment
Source: Front Cell Infect Microbiol. 2025 Nov 19;15:1681781. doi: 10.3389/fcimb.2025.1681781 (PMC12672516; doi:10.3389/fcimb.2025.1681781)
Supplement: Supplementary file 1 [file Supplementaryfile1.docx]

**Supplemental Material

Supplemental Methods**

**Oral Health Data Collection**During the first week of their hospital stay, patients received a full oral exam from the National Institute of Dental and Craniofacial Research dental team at the Clinical Center (1). Two assessments were performed: one measured tooth health using the Decayed, Missing, and Filled Teeth (DMFT) index, and the other evaluated gum health (1). Gum disease was first classified as none, mild, moderate, or severe based on clinical attachment loss and the depth of the pockets around the gums (periodontal pocket depth) (1).

**Oral and Gut Microbiome Sample Collection and Processing**
Oral and stool microbiome samples were collected and processed using standardized, previously published protocols (1, 2). Stool samples were obtained daily during the first week of admission and once per week thereafter for the four-week inpatient treatment. Immediately following collection, samples were transferred to −20°C within 15 minutes and held for no more than three days prior to whole stool homogenization and aliquoting. Homogenization involved dilution in phosphate-buffered saline, blending with a Stomacher circulator, and filtration before long-term storage at −80°C for downstream DNA extraction and sequencing. See the primary gut microbiome paper for full details of the processing procedure (2).

Oral samples were collected from the tongue dorsum via sterile tongue brushings up to 10 times across the 4-week inpatient treatment period. Samples were vortexed, centrifuged, and pelleted prior to −80°C storage. DNA was extracted and the 16S rRNA gene was amplified using the Ion 16S™ Metagenomics Kit (ThermoFisher), targeting seven hypervariable regions (V2, V3, V4, V6-7, V8, V9), with sequencing performed on the Ion Torrent S5 XL platform. All sequencing and quality control procedures, including mock community standards and read count thresholds, are described in full in the primary studies; for detailed sequencing and bioinformatics protocols, see previous publications (1, 2) .

**Bioinformatics Sample Pre-Processing and Oral and Gut Data Merging**Raw 16S rRNA gene sequence data from oral and stool samples were processed following previously published pipelines(1, 2) For both sample types, sequencing was performed using the Ion Torrent S5 platform and processed using the USEARCH v11 pipeline (3). Reads were filtered, dereplicated, and clustered into OTUs (gut) or denoised into zero-radius OTUs (ZOTUs, oral) after quality trimming and primer removal. Taxonomy was assigned using the SINTAX algorithm with the RDP v16 reference database. OTU/ZOTU tables were generated for each hypervariable region (V2, V3, V4, V6-7, V8), summarized at the genus level, and merged by genus across all V regions. To combine data across regions, reconstructed counts were calculated using the root mean square of genus-level counts across V regions. To prepare for the current within patient cross-site microbiome analysis, each reconstructed count table (oral and gut) was filtered to retain only genera present in ≥25% of samples within each dataset, resulting in 144 oral and 67 gut genera. The two filtered datasets were then merged using an outer join by unique genus, yielding a combined table of 159 genera for analysis. Average counts across all timepoints for each sampling site over all participants is shown in **Supplemental Table 2**.

**Oral and Gut Microbiome Alpha Diversity Calculation**The Shannon Diversity Index (SDI) was computed for both oral and gut genera at each timepoint*patient combination using the vegan package function “diversity” and validated by manual computation using the following equation, for n genera present within a sample type, where pi is the proportion of the summed count values for each genus:

$$\sum_{i=1}^{n} p_{i}*logp_{i}$$

**Data Transformation and Distance Metrics for Within-Participant Oral–Gut Similarity**
To assess within-participant microbiome similarity, we began with a matrix of genus-level oral and gut count data for each patient at each timepoint, restricted to timepoints where paired oral and gut samples were available. All missing genus counts were set to zero to indicate absence.

The Sørensen–Dice index was calculated for each patient–timepoint combination as a measure of shared microbial presence and was calculated by dividing twice the number of shared oral and gut genera by the sum of the number of oral and gut genera at each timepoint. A genus was considered "present" if its count exceeded zero (4). Next, a centered log-ratio (CLR) transformation was applied to the entire dataset using the getTableMeans() function from the *deleuze* package (https://github.com/thomazbastiaanssen/deleuze). This function applies an offset to ensure that the logarithmic transformation is defined (i.e., this avoids taking the log of a zero value).

Using the CLR transformed data, we computed both the Robust Aitchison distances as follows (5). First, after obtaining the CLR transformed data we positionally set any cells with a pre-transformation value of 0 to missing (**NA**) so that the robust distance calculation would only be computed using genera present in both oral and gut at each timepoint*patient combination. Then, for each genus at each timepoint*patient combination, the squared difference between the gut and oral CLR-transformed count values was computed. Note that for the Robust Aitchison, with original 0s set to NA, the squared difference terms are only computed for genera present in both oral and gut at each timepoint, whereas the standard Aitchison would include squared differences between all pairs in which either oral or gut was present. Finally, the squared difference terms were summed within each timepoint*patient combination to obtain a Robust Aitchison distance for each patient at each timepoint.

**Testing Associations and Overlap with Past Drinking Behaviors**Previous work identified variables that affected the oral and gut microbial diversity in this cohort of individuals with alcohol use disorder. We previously showed that alcohol choice type (beer, wine, liquor, or beer and liquor) had an overall effect on the oral microbiome (1) and likewise, we previously showed that alcohol dose as described as “very heavy drinkers (VHD)” or “less heavy drinkers (LHD)” had an effect on the gut microbial diversity (2). Categorization of alcohol dosing type is defined as LHD, <10 drinks/d and very heavy drinkers VHD, 10 or more drinks/d. To explore how these variables might affect the overlap and changes observed in this exploratory analysis, we used generalized linear mixed models (LMM) to look at how alcohol choice and dose (LHD/VHD) were associated with overlap metrics (Sorenson-Dice, number of shared genera, Robust Aitchison Distance) during the first week. For the number of shared genera, which is a count variable, we used a Poisson LMM.

**Supplemental Tables

Supplemental Table 1: Sample Population Demographics**

| **Demographics (n=22)** | **Mean ± SD, (range) or n (%)** |
| --- | --- |
| Age | 45.82±13.0 |
| Gender | Male: 14 (63.6%)  Female: 8 (63.4%) |
| Race | White:13 (59.1%)  Black: 6 (27.3%)  Multiple: 2 (9.1%)  Unknown:1 (4.5%) |
| Body Mass Index kg/m^2^ | 23.87±2.55, (19.0-29.0) |
| Smoker Status | Yes: 16 (72.72%)  No: 6 (27.28%) |
| Periodontal Assessment | No periodontitis: 3 (13.6%)  Mild periodontitis: 2 (9.1%)  Moderate periodontitis:14 (63.6%)  Severe periodontitis: 3 (13.6%) |
|  | |
| **90-day Timeline Followback**  **(n = 21)** | **Mean ± SD, (range)** |
| Drinking days | 81.38 ± 15.21, (29–90) |
| Average drinks/day | 16.20 ± 10.59, (3.3–40) |
| Total drinks over 90 days | 1457.56 ± 952.82, (299.3–3600) |
| Heavy drinking days | 76.57 ± 22.51, (3–90) |
| Days since last drink (range) | -1.59 ± 4.85 |

**Supplemental Table 2: Average over participant of oral and gut genus level counts for all timepoints. [Supplemental_Table_2.csv]**

**Supplemental Table 3: Average Shannon diversity index association between oral and gut microbiome at each timepoint**

| **Days/timepoint** | **DF** | **t Ratio** | **Prob < t** |
| --- | --- | --- | --- |
| 1 | 3.218 | -3.45 | **0.018** |
| 2 | 27.983 | -3.39 | **0.001** |
| 3 | 28.455 | -3.15 | **0.002** |
| 4 | 30.343 | -4.58 | **<.001** |
| 5 | 28.347 | -4.57 | **<.001** |
| 6 | 22.498 | -4.05 | **<.001** |
| 7 | 27.843 | -4.80 | **<.001** |
| 8 | 30.358 | -4.56 | **<.001** |
| 15 | 32.883 | -4.55 | **<.001** |
| 22 | 31.484 | -5.90 | **<.001** |
| Overall timepoints | 36.817 | 6.011 | **<.001** |

Student’s t-test assessing oral and gut microbiome Shannon diversity index at each timepoint. Bold indicates p<.05.

**Supplemental Table 4: Typical Alcohol Choice and Dose Type**

|  | **Level** | **Overall** |
| --- | --- | --- |
|  |  | n=22 (%) |
| Drinker Type | LHD | 8 (36.4%) |
|  | VHD | 14 (63.6%) |
| Typical Alcohol | Beer | 4 (18.2%) |
|  | Beer and Liquor | 5 (22.7%) |
|  | Liquor | 6 (27.3%) |
|  | Wine | 7 (31.8%) |

Abbreviations: LHD: less heavy drinker; VHD: very heavy drinker

**Supplemental Table 5: Associations of genera overlap by drink dose and type**

|  | **Genera in common per timepoint per PID** | | | ***Robust Aitchison*** | | | **Log (Sorenson-Dice)** | | |
| --- | --- | --- | --- | --- | --- | --- | --- | --- | --- |
|  | IRR | CI | **P** | Estimate | CI | **P** | Estimate | CI | **P** |
| (**Int**) | 10.18 | 8.62 – 12.02 | **<0.001** | 13.16 | 11.71 –14.61 | **<0.001** | -1.51 | -1.63 – -1.38 | **<0.001** |
| Drinker  Type [VHD] | 1.25 | 1.07 – 1.47 | **0.006** | 0.04 | -1.29 –1.36 | 0.958 | 0.15 | 0.03 –0.26 | **0.013** |
| Choice [Beer and Liquor] | 0.84 | 0.68 – 1.02 | 0.084 | -1.14 | -2.93 –0.66 | 0.212 | -0.17 | -0.33 – -0.02 | **0.030** |
| Choice [Liquor] | 0.76 | 0.62 – 0.92 | **0.006** | -1.28 | -3.04 –0.47 | 0.150 | -0.25 | -0.40 – -0.09 | **0.002** |
| Choice [Wine] | 0.74 | 0.60 – 0.92 | **0.006** | -1.22 | -2.97 –0.53 | 0.168 | -0.26 | -0.42 – -0.11 | **0.001** |
| **Random Effect** |  | | | | | | | | |
| σ^2^ | 0.10 | | | 5.01 | | | 0.07 | | |
| τ_00_ _PID_ | 0.00 | | | 0.76 | | | 0.00 | | |
| ICC | 0.05 | | | 0.13 | | | 0.00 | | |
| N _PID_ | 22 | | | 22 | | | 22 | | |
| Observations | 100 | | | 100 | | | 100 | | |
| Marginal R^2^ / Conditional R^2^ | 0.192 / 0.228 | | | 0.041 / 0.167 | | | 0.179 / 0.180 | | |

Footnote: p-values computed using Wald tests for the predictors in each LMM; drinker type LHD and alcohol choice of beer were the reference categories. Abbreviations: IRR: Incidence rate ratios; CI: Confidence interval; P: p-value; VHD: Very heavy drinker; LHD: less heavy drinker. Bold indicates p<.05.

**Supplemental Table 6: Associations of overlap by previous drinking metrics**

|  | **Genera in common per timepoint per PID** | | | **Robust Aitchison** | | | **Log (Sorenson-Dice)** | | |
| --- | --- | --- | --- | --- | --- | --- | --- | --- | --- |
| *Predictors* | *IRR* | *CI* | *p* | *Estimate* | *CI* | *p* | *Estimate* | *CI* | *p* |
| (Intercept) | 8.74 | 7.35 – 10.39 | **<0.001** | 12.55 | 11.39 – 13.71 | **<0.001** | -1.67 | -1.82 – -1.52 | **<0.001** |
| Avg Drinks/d | 1.01 | 1.00 – 1.02 | 0.096 | -0.01 | -0.07 – 0.05 | 0.776 | 0.00 | -0.00 – 0.01 | 0.188 |
| Days Since Last Drink | 1.00 | 0.97 – 1.04 | 0.767 | 0.06 | -0.14 – 0.25 | 0.553 | -0.00 | -0.03 – 0.02 | 0.775 |
| Avg Drinks/d Ã— Days Since Last Drink | 1.00 | 1.00 – 1.01 | 0.605 | 0.01 | -0.02 – 0.04 | 0.646 | 0.00 | -0.00 – 0.00 | 0.565 |
| **Random Effects** |  | | | | | | | | |
| σ^2^ | 0.10 | | | 5.01 | | | 0.07 | | |
| τ_00_ _PID_ | 0.02 | | | 0.67 | | | 0.01 | | |
| ICC | 0.15 | | | 0.12 | | | 0.17 | | |
| N _PID_ | 22 | | | 22 | | | 22 | | |
| Observations | 100 | | | 100 | | | 100 | | |
| Marginal R^2^ / Conditional R^2^ | 0.093 / 0.227 | | | 0.042 / 0.154 | | | 0.040 / 0.203 | | |

Footnote: p-values computed using Wald tests for the predictors in each LMM. Abbreviations: IRR: Incidence rate ratios; CI: Confidence interval; p: p-value. Bold indicates p<.05.

**Supplemental Figures**

**Supplemental Figure 1: The number of oral and gut microbiome shared genera decreased with abstinence from alcohol**

**
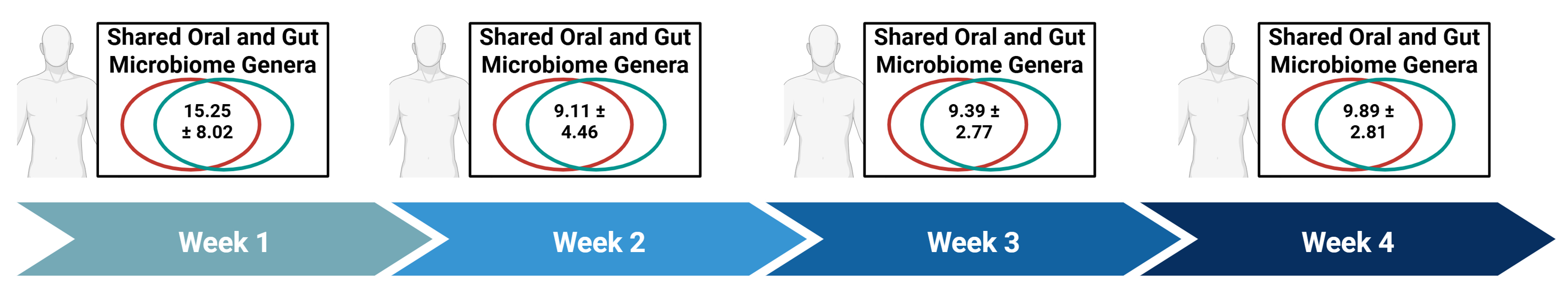
**

Legend: The number of shared oral and gut microbiome genera across participants at each week during inpatient treatment and early abstinence from alcohol.

**Supplemental Figure 2: Assessment of Shannon diversity index between oral and gut microbiome at each sampling timepoint**


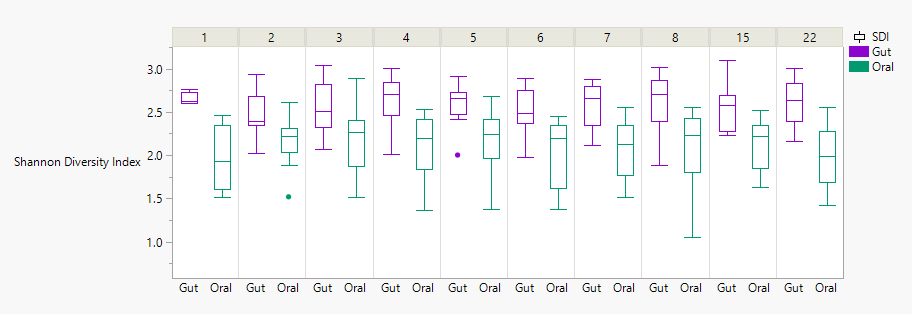


Legend: Box plot of Shannon Diversity Index (y-axis) each oral and gut microbiome (x-axis) sample at each timepoint for all participants.

**References**

1. Barb JJ, Maki KA, Kazmi N, Meeks BK, Krumlauf M, Tuason RT, et al. The oral microbiome in alcohol use disorder: a longitudinal analysis during inpatient treatment. J Oral Microbiol. 2022;14(1):2004790.

2. Ames NJ, Barb JJ, Schuebel K, Mudra S, Meeks BK, Tuason RTS, et al. Longitudinal gut microbiome changes in alcohol use disorder are influenced by abstinence and drinking quantity. Gut Microbes. 2020;11(6):1608-31.

3. Edgar RC. Search and clustering orders of magnitude faster than BLAST. Bioinformatics. 2010;26(19):2460-1.

4. Zou KH, Warfield SK, Bharatha A, Tempany CM, Kaus MR, Haker SJ, et al. Statistical validation of image segmentation quality based on a spatial overlap index. Acad Radiol. 2004;11(2):178-89.

5. Martino C, Morton JT, Marotz CA, Thompson LR, Tripathi A, Knight R, et al. A Novel Sparse Compositional Technique Reveals Microbial Perturbations. mSystems. 2019;4(1).
